# Research Trends and Thematic Evolution of *Salvadora persica* (Miswak) in Oral Health: A Bibliometric Analysis

**DOI:** 10.1155/ijod/9050182

**Published:** 2026-08-19

**Authors:** Norfaezah Ahmad, Muhammad Ashraf Fauzi, Nur Armidha Nadihah, Nurulhasanah Mustapar, Sharifah Nurul Akilah Syed Mohamad, Mohd Hafiz Arzmi

**Affiliations:** ^1^ Department of Prosthodontics, Kulliyyah of Dentistry, International Islamic University Malaysia, Bandar Indera Mahkota, Kuantan 25200, Pahang Darul Makmur, Malaysia, iium.edu.my; ^2^ Department of Basic Medical Sciences, Kulliyyah of Medicine, International Islamic University Malaysia, Kuantan, Pahang, Malaysia, iium.edu.my; ^3^ Faculty of Industrial Management, Universiti Malaysia Pahang Al-Sultan Abdullah, Lebuh Persiaran Tun Khalil Yaakob, Gambang 26300, Pahang, Malaysia, umpsa.edu.my; ^4^ Graduate Engineering and Technological Business School, Universiti Malaysia Pahang Al-Sultan Abdullah, Kuantan, Malaysia, umpsa.edu.my; ^5^ Centre for Advanced Industrial Technology, Universiti Malaysia Pahang Al-Sultan Abdullah, Pekan 26600, Pahang, Malaysia, umpsa.edu.my; ^6^ Department of Fundamental Dental and Medical Sciences, Kulliyyah of Dentistry, International Islamic University Malaysia, Bandar Indera Mahkota, Kuantan 25200, Pahang Darul Makmur, Malaysia, iium.edu.my; ^7^ Cluster of Cancer Research Initiative IIUM (COCRII), International Islamic University Malaysia, Kuantan, Pahang, Malaysia, iium.edu.my; ^8^ Melbourne Dental School, The University of Melbourne, Victoria, Australia, unimelb.edu.au

**Keywords:** antimicrobial activity, bibliometric analysis, miswak, oral hygiene, *Salvadora persica*

## Abstract

**Aim:**

This study aimed to map the publication landscape, intellectual structure and thematic organisation of *Salvadora persica* (miswak) research in oral health using bibliometric analysis.

**Methods:**

The Web of Science (WoS) Core Collection was used to retrieve publications. Search terms were pre‐determined on *S. persica* and oral health. The performance assessment was based on publication trends, high‐productivity authors and countries, and the most‐cited articles. Co‐citation, bibliographic coupling and keyword co‐occurrence analysis were carried out using VOSviewer to visualise intellectual structures and research themes.

**Findings:**

A total of 295 journal articles were included in the final dataset, with 3035 citations in total and 2795 excluding self‐citations. The number of research outputs has grown significantly since 2014. The major geographical contributors were Saudi Arabia, India and Egypt. Co‐citation analysis showed that the intellectual structure of the field is anchored in references related to preventive dentistry, oral hygiene practices, periodontal health, phytochemistry and antimicrobial research. Four significant thematic groups were identified through bibliographic coupling and keyword searches: antimicrobial and antioxidant mechanisms; behavioural and preventive oral health practices; extract‐based and clinical applications in dental caries management; and plaque control and gingival health outcomes. The clusters indicate that the literature is organised around traditional oral hygiene use, antimicrobial and phytochemical mechanisms, clinical oral health outcomes and natural product applications.

**Conclusion:**

The evidence base for *Salvadora persica* has evolved into a structured, multidisciplinary field. The literature increasingly frames miswak as a potential adjunct to conventional oral hygiene; however, bibliometric findings alone cannot establish clinical effectiveness.

## 1. Introduction

Oral disease continues to be the most prevalent non‐communicable global condition affecting billions each year. Oral disease substantially burdens individual quality of life, the health system, and the economy [1]. Dental caries and periodontal disease are primarily attributable to biofilm accumulation, dysbiosis of the oral microbiome, and poor plaque control [1–3]. While conventional oral hygiene strategies such as toothbrushing and fluoride use have been effective, their uptake and effectiveness in marginalised and low‐income communities are limited by cost, income inequality, cultural factors and poor compliance [1, 2, 4]. Current efforts to control oral disease are being implemented across diverse populations, with a focus on culturally and demographically centred approaches. Some of the strategies involve mechanical cleaning combined with specific delivery of bioactive agents [1–3].

Plant‐based oral hygiene devices are once again of interest within this wider preventive landscape. The World Health Organisation has approved the use of traditional chewing sticks, especially in communities where they are practised and effective, which is relevant to prevention, community involvement and proper use of technology to meet the primary care goals [1, 3]. One of the most common oral hygiene products is the traditional chewing stick, *Salvadora persica* (also known as miswak), used in Africa, the Middle East and Asia, with a deep historical, cultural, and religious background [5–7]. Narrative and systematic reviews have shown that the miswak removes plaque both mechanically and biochemically. Moreover, it prevents and treats a variety of oral diseases, such as periodontal disease, thanks to its antibacterial, anti‐plaque, anti‐cariogenic, anti‐fungal, antioxidant, anti‐inflammatory and analgesic properties [1, 2, 7]. When used appropriately, the miswak and products containing its extracts have the potential to be as effective as conventional oral hygiene products in controlling plaque and gingivitis [2–4, 8].

*S. persica* has the phytochemical profiles of benzyl isothiocyanate, benzyl nitrile, essential oils, saponins, flavonoids, sterols, tannins, and organic acids. This rich profile has been linked to its antimicrobial, anti‐carcinogenic, anti‐gingivitis and salivary‐modulating effects [1, 3, 6, 8, 9]. Consequently, numerous formulations have been designed with the help of these bioactive constituents, such as chewing sticks, dentifrices, mouthwashes, chewing gums and local delivery systems, with the evidence starting to appear on their effectiveness in the form of plaque scores, salivary pH, bacterial counts, gingival indices, and wound healing responses [1, 2] In addition to dentistry, *S. persica* has been explored due to its various pharmacological features and industrial uses [6, 8, 9]. Nevertheless, current studies on *S. persica* have focused on product development, phytochemistry, in vitro assays, animal models and clinical trials [10–13]. Current reviews are largely narrative or scoping in nature, emphasising therapeutic effects and traditional uses rather than quantitative mapping of the evolution, structure and thematic clustering of the research field [3, 4, 6–8].

Here, we identify a clear knowledge gap through a systematic bibliometric analysis that characterises *S. persica* research in oral health. Publication trends, authors, institutions and collaborations with an interest in this plant, core journals, and the link between therapeutic mechanisms and clinical applications are evidently missing from the literature. Other research gaps highlighted in previous reviews include standardisation of extracts, optimisation of dosage and delivery systems, long‐term safety and translation of mechanistic insights into robust clinical protocols [1–4, 6, 8, 9].

The current study maps the Web of Science (WoS)‐indexed literature on *S. persica* in oral health and identifies major publication patterns, research orientations and intellectual structures and gaps. This bibliometric analysis does not assess clinical effectiveness, guideline quality or treatment efficacy. Instead, it provides a structured overview of how the field is represented in the indexed literature and highlights areas requiring further methodological, experimental and clinical development.

## 2. Methodology

### 2.1. Bibliometric Approach

Bibliometric analysis is a quantitative research methodology that examines large bibliographic databases using scientometric techniques to describe research landscapes systematically [14]. It offers more informative and broad perspectives on research development and impact, as well as systematic reviews and meta‐analyses [15].The two major components in this approach are: 1. Performance analysis, which evaluates the contributions of key research entities, including authors, journals, institutions, and countries, and 2. Science mapping, which synthesises existing knowledge to reveal the intellectual structure, thematic organisation, and evolution of a research field. Publication counts are used in performance analysis to indicate productivity, whereas citation metrics measure the impact. In the present study, science mapping was used to understand the research development posts on *S. persica*. Specifically, bibliographic coupling was used to identify current and emerging research themes, while co‐word analysis was applied to explore thematic relationships and potential future research directions.

### 2.2. Research Design and Data Collection Procedures

A predefined search string using relevant keywords ((“*Salvadora persica*” OR siwak OR Miswak) AND (“oral health” OR “oral appl ^∗^” OR “oral hygien ^∗^” OR perio ^∗^ OR oral ^∗^)) (Table 1) was used to retrieve related publications. Searches were conducted using the WoS Topic Search (TS) function within the WoS Core Collection. The WoS Core Collection was selected as the sole database because it provides a curated and standardised source of bibliographic records, citation data and cited references suitable for bibliometric analysis. Its structured metadata enables the consistent extraction of publication information, citation counts, author details, source titles, and reference lists, which are required for performance analysis, co‐citation analysis, bibliographic coupling and keyword co‐occurrence mapping. In addition, WoS records are highly compatible with VOSviewer, allowing the reliable construction of science‐mapping networks. Therefore, the use of the WoS Core Collection was considered appropriate for generating a reproducible and internally consistent bibliometric dataset. Only the peer‐reviewed journal articles were included; books, book chapters, conference proceedings and editorials were excluded to ensure the quality and consistency of the retrieved studies, a common approach in bibliometric studies.

**Table 1:** Search string in WoS database.

No.	Keywords	Justification
1	“*Salvadora persica*” OR siwak OR Miswak	To comprehensively capture the botanical name used across different disciplines and regions.
2	“oral health” OR “oral appl ^∗^” OR “oral hygien ^∗^” OR perio ^∗^ OR oral ^∗^	To capture the full scope of dental and periodontal research contexts, including preventive, clinical and hygiene‐related applications.

*Note*:  ^∗^ The asterisk indicates truncation, was used to retrieve different word endings.

### 2.3. Data Screening, Eligibility Criteria and Bibliometric Processing

The search was conducted in the WoS Core Collection on 16 January 2026 using the TS field. WoS was selected because it provides standardised bibliographic metadata, citation information and cited references suitable for bibliometric mapping and network analysis. The retrieved records were exported in plain‐text format using the “Full Record and Cited References” option to ensure compatibility with VOSviewer. Before the analysis, the dataset was screened to ensure relevance to oral and dental health. Titles, abstracts and keywords were reviewed to confirm that each record focused on *S. persica*, miswak or siwak in relation to oral health, dentistry, oral hygiene, periodontal health, oral microbiology, dental caries, oral biofilms or dental‐material applications. Records that focused on non‐oral or non‐dental uses, systemic pharmacology unrelated to oral health, agricultural or botanical studies without dental relevance and non‐article document types were excluded. Duplicate records were checked manually using DOI, article title, author name and publication year.

Bibliometric analysis and network visualisation were performed using VOSviewer version 1.6.20. The analyses included citation analysis, co‐citation analysis, bibliographic coupling and keyword co‐occurrence analysis. Full counting was applied, whereby each publication, citation or keyword occurrence was given equal weight in the analysis. Association‐strength normalisation was used to construct and visualise the bibliometric networks. Thresholds for inclusion in each network were determined before the final map generation to balance analytical coverage and visual clarity. These thresholds were applied to reduce network overcrowding while retaining the most influential and relevant items for interpretation. In the visualisation maps, node size represents the frequency or weight of an item, link thickness indicates the strength of the relationship between items, and node colour represents clusters automatically generated by VOSviewer based on the relatedness of items within the network.

**Figure 1:**
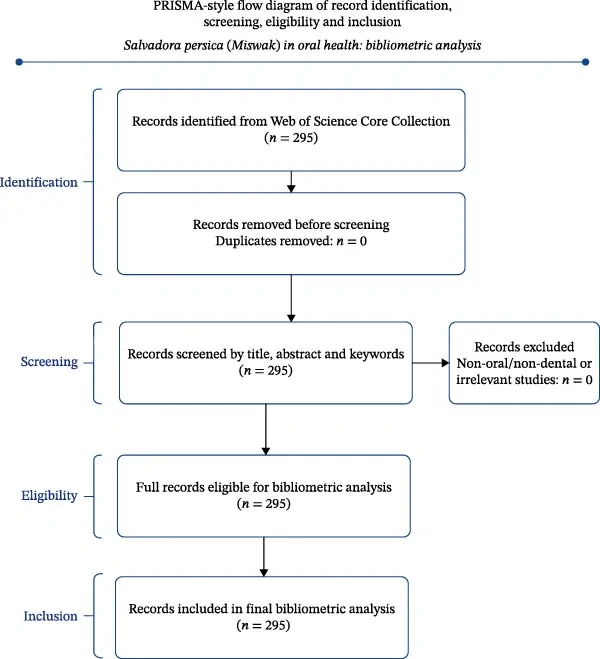
PRISMA‐style flow diagram of record identification, screening, eligibility and inclusion for the bibliometric analysis.

To improve transparency in the selection process, a PRISMA‐style flow diagram was used to summarise the identification, screening, eligibility and inclusion of records for the bibliometric analysis (Figure 1). Although this study is a bibliometric rather than a systematic review, the flow diagram was adapted to clearly report how records were retrieved from the WoS Core Collection, screened for relevance to oral and dental medicine, and included in the final analytical dataset. The final dataset was then used for performance analysis and VOSviewer‐based science mapping.

## 3. Findings and Discussion

The search was conducted in the WoS Collection on 16 January 2026 using the final predefined search strategy. The final dataset comprised 295 journal articles, which had 3035 citations and 2795 citations, excluding self‐citations. No duplicate records were identified, and all eligible records were included in the bibliometric analysis.

### 3.1. Annual Publication Growth Trend

**Figure 2:**
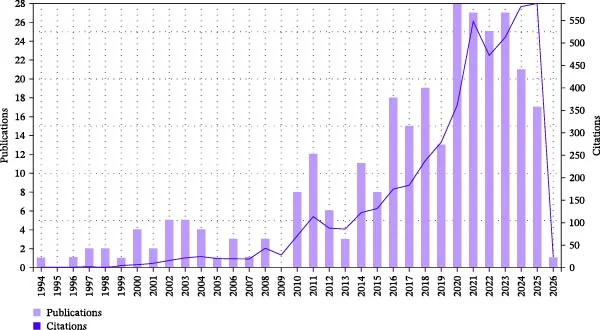
Annual publication output of *Salvadora persica* in oral health research.

The development of *S. persica* oral health studies is evident and steady, which is a sign of the maturity of this field. The mid‐1990s to the early 2000s (Figure 2) had few publications, primarily exploratory, which documented a simple antimicrobial effect and plaque control. However, despite the importance of these publications in sparking scientific interest, these early studies had relatively limited citation visibility [16]. The increase in publications and citations was slow between 2005 and 2013, and it was linked to the fact that more people started accepting the use of plant‐based oral care agents and better experimental design. A study was initiated to examine the relationship between *S. persica* and biofilm inhibition, periodontal inflammation, and the number of cariogenic bacteria. There was a specific acceleration in the field around 2014 when the number of publications and citations per year increased significantly. This has been coupled with a rise in interest in natural therapeutics and antimicrobial resistance worldwide, and, as a result, an increase in interest in adding *S. persica* to modern oral health products, such as toothpastes and mouth rinses.

Citation activity was most active between 2020 and 2024, which indicates high publication visibility and impact. This was credited to a better mechanistic understanding and clinically significant results. Nevertheless, there is a noticeable decrease in citations over the last few years, which, though it may be interpreted as a reduction in interest in the study, can be explained by the citation lag. Overall, the trend in annual publications suggests a clear shift from early, more descriptive studies to research on translational and clinical outcomes.

### 3.2. Most Productive Authors and Countries

**Figure 3:**
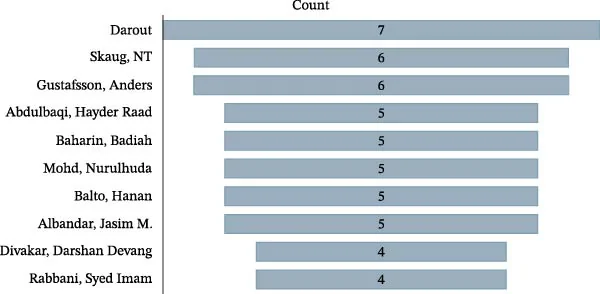
Leading authors and their publication output in *Salvadora persica* oral health research.

It was found that scholarly contributions in the domain of oral health research on *S. persica* were relatively evenly distributed. There was no dominant author in terms of authorship (Figure 3). Darout had the highest productivity, with seven publications (2.37% of the total dataset); Skaug and Gustafsson, six publications (2.03%); Abdulbaqi, Baharin, Mohd, Balto, and Albandar, five publications (1.70%); and, lastly, Divakar and Rabbani, four publications (1.36%). The top 10 authors accounted for a small proportion of total publications, suggesting a wide range of interests across research groups and, consequently, a collaborative, multidisciplinary research environment rather than a focus on individuals or groups.

**Figure 4:**
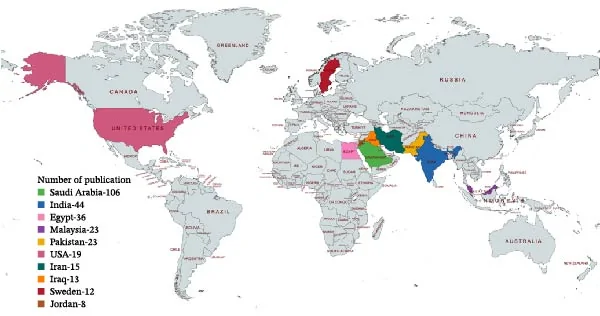
Most productive countries in *Salvadora persica* oral health research.

Nevertheless, the country‐level analysis found Saudi Arabia as the most contributing country in the research on *S. persica* oral health, with 106 publications. India ranked second with 44 publications and Egypt ranked third with 36, as shown in Figure 4. The localisation of studies in these geographical areas indicates the culturally relevant and clinically applicable application of *S. persica* in these areas. Outside the Middle East and South Asia, Malaysia and Pakistan had 23 publications each, and the United States (19) and Iran (15) had also produced a large number of publications. Publications were also published in Iraq, Sweden, and Jordan, indicating that this field of study had an international scope. The research distribution shows that *S. persica* research is clustered in countries that have traditionally been linked to the use of the product as well as in countries interested in research on preventive dentistry and oral hygiene.

### 3.3. Highly Cited Publication and Citation Impact

Table 2 shows that the most cited study was by Sofrata et al. [17], who reported that benzyl isothiocyanate exhibited high antibacterial activity against Gram‐negative bacteria in *S. persica*. This was preceded by Wu et al. [18], who provided a summary of chewing sticks as natural oral hygiene instruments and laid the scientific foundation for understanding traditional practices in current periodontal studies. Subsequently, the narrative reviews by Halawany [7] and Haque and Alsareii [3] summarised the available information on the impact of *S. persica* on plaque control and gingival health,and were among the most referenced.

**Table 2:** The most highly cited studies on *Salvadora persica* in oral health.

Authors	Title	Publication year	Source title	Cited by
Sofrata et al. [17]	Benzyl Isothiocyanate a Major Component from the Roots of *Salvadora Persica* Is Highly Active against Gram‐Negative Bacteria	2011	Plos One	150
Wu et al. [18]	Chewing sticks: timeless natural toothbrushes for oral cleansing	2001	Journal of Periodontal Research	118
Halawany [7]	A review on miswak (*Salvadora persica*) and its effect on various aspects of oral health	2012	Saudi Dental Journal	114
Noumi et al. [19]	Antifungal properties of *Salvadora persica* and *Juglans regia* L. extracts against oral Candida strains	2010	European Journal of Clinical Microbiology & Infectious Diseases	100
Haque and Alsareii [3]	A review of the therapeutic effects of using miswak (*Salvadora Persica*) on oral health	2015	Saudi Medical Journal	95
Hochli et al. [20]	Interventions for orthodontically induced white spot lesions: a systematic review and meta‐analysis	2017	European Journal of Orthodontics	92
A. H. Sofrata et al. [21]	Strong antibacterial effect of miswak against oral microorganisms associated with periodontitis and caries	2008	Journal of Periodontology	86
Aumeeruddy et al. [6]	A review of the traditional and modern uses of *Salvadora persica* L. (Miswak): Toothbrush tree of Prophet Muhammad	2018	Journal of Ethnopharmacology	82
Muthee et al. 2011 [22]	Ethnobotanical study of anthelmintic and other medicinal plants traditionally used in Loitoktok district of Kenya	2011	Journal of Ethnopharmacology	81
Moghadam et al. [23]	Current herbal medicine as an alternative treatment in dentistry: In vitro, in vivo and clinical studies	2020	European Journal of Pharmacology	80

More recent, highly cited research focuses on the microbiological specificity, expanded therapeutic use and clinical applicability of *S. persica*. These are studies by Noumi et al. [19], who have reported antifungal activity against oral Candida species; Sofrata et al. [21], who have reported antimicrobial activity against caries‐ and periodontitis‐causing microorganisms; and Aumeeruddy et al. [6], who have linked the traditional knowledge to the modern pharmacological evidence. In the meantime, Hochli et al. [20] and Moghadam et al. [23] published systematic reviews that considered the field of herbal dentistry more broadly and included studies on *S. persica* as part of evidence‐based dentistry. Collectively, the numerous publications referenced suggest a desire and a shift in *S. persica* research toward thorough reviews and research with a clinical angle.

### 3.4. Co‐Citation Analysis

**Figure 5:**
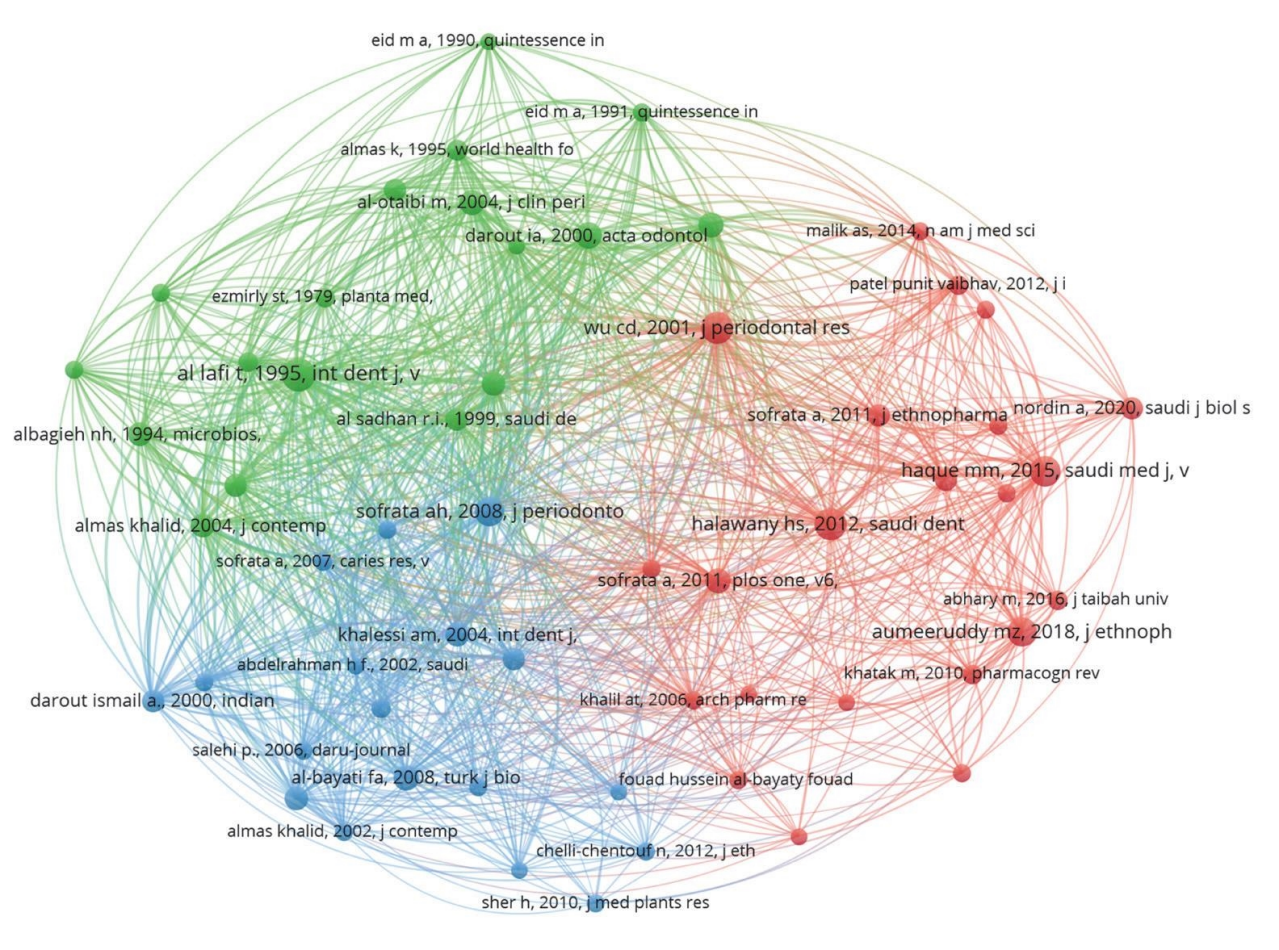
Co‐citation network of influential cited references in *Salvadora persica* oral health research.

The 15 cited references included in the co‐citation network were not manually selected (Figure 5). They were identified by VOSviewer after applying a minimum co‐citation threshold of 58. Co‐citation analysis examines how often two references are cited together by the documents in the dataset. References that met the threshold were included in the network because they represented the most frequently co‐cited sources within the final dataset. Three distinct clusters were identified, with strong internal connectivity and significant cross‐linkages, indicating the evolution of the field through interlinkages among basic oral health research, ethnopharmacology and modern pharmacology, and clinical dental studies. This framework outlines how early periodontal and public health studies contributed to subsequent research, thereby laying a strong foundation for current oral hygiene knowledge and practice.

#### 3.4.1. Cluster 1 (Green): Foundational Oral Health and Periodontal Literature

The first cluster comprises the literature on general oral health, preventive dentistry and periodontal health. Literature in this cluster provides examples, such as epidemiological studies, indices and global oral health frameworks, that enable the assessment of oral hygiene, gingival health and disease prevalence. The central location of this cluster in the network and its co‐citation frequency indicate that foundational oral health and periodontal literature supported the contextualisation of *S. persica* clinical and experimental studies within the broader dental health field.

#### 3.4.2. Cluster 2 (Blue): Early Clinical and Observational Studies on Miswak Use

The second cluster comprises literature on observational and clinical studies of *miswak*. Important outputs include caries prevalence, periodontal status and oral hygiene outcomes. Cross‐sectional surveys, comparative studies and early clinical trials constitute the bulk of the references in this cluster. A clear transition from basic oral health concepts to the application of *S. persica* as a functional oral hygiene tool is noted. The dense interconnections suggest the contribution of these studies to the early clinical understanding of the relevance of traditional oral hygiene practices in contemporary times.

#### 3.4.3. Cluster 3 (Red): Pharmacological, Phytochemical and Mechanistic Research

This cluster comprises pharmacological and ethnopharmacological literature, covering topics including phytochemical profiling, bioactive compounds and antimicrobial activity. Co‐citations in this cluster include natural pharmacology and medicine as well as experimental biology. Clinical observations are elucidated at the mechanistic level, linking specific chemical constituents in the plant to bioactivity, specifically antimicrobial and antibiofilm activities. The strong connectivity to the clinical cluster reflects the integration of laboratory evidence with oral health applications at this stage.

The co‐citation map shows that the intellectual structure of *S. persica* oral health research is organised around three related knowledge bases: foundational oral health and periodontal literature, clinical and observational studies of miswak use and pharmacological or phytochemical research. Because this analysis does not include a temporal modelling component, the clusters should be interpreted as intellectual groupings rather than evidence of a chronological progression.

### 3.5. Bibliographic Coupling Analysis

Bibliographic coupling analysis highlights the intellectual connections among studies through shared references, revealing existing themes and emerging areas of research [14]. The citation network reveals thematic clusters, key translational studies, and the organisation of the literature. Results of this analysis demonstrate the evolution of research from basic to applied/clinical and highlight the balance achieved between mechanistic and application‐oriented studies. This analysis also identifies meaningful research gaps and opportunities in oral hygiene and preventive dentistry.

**Figure 6:**
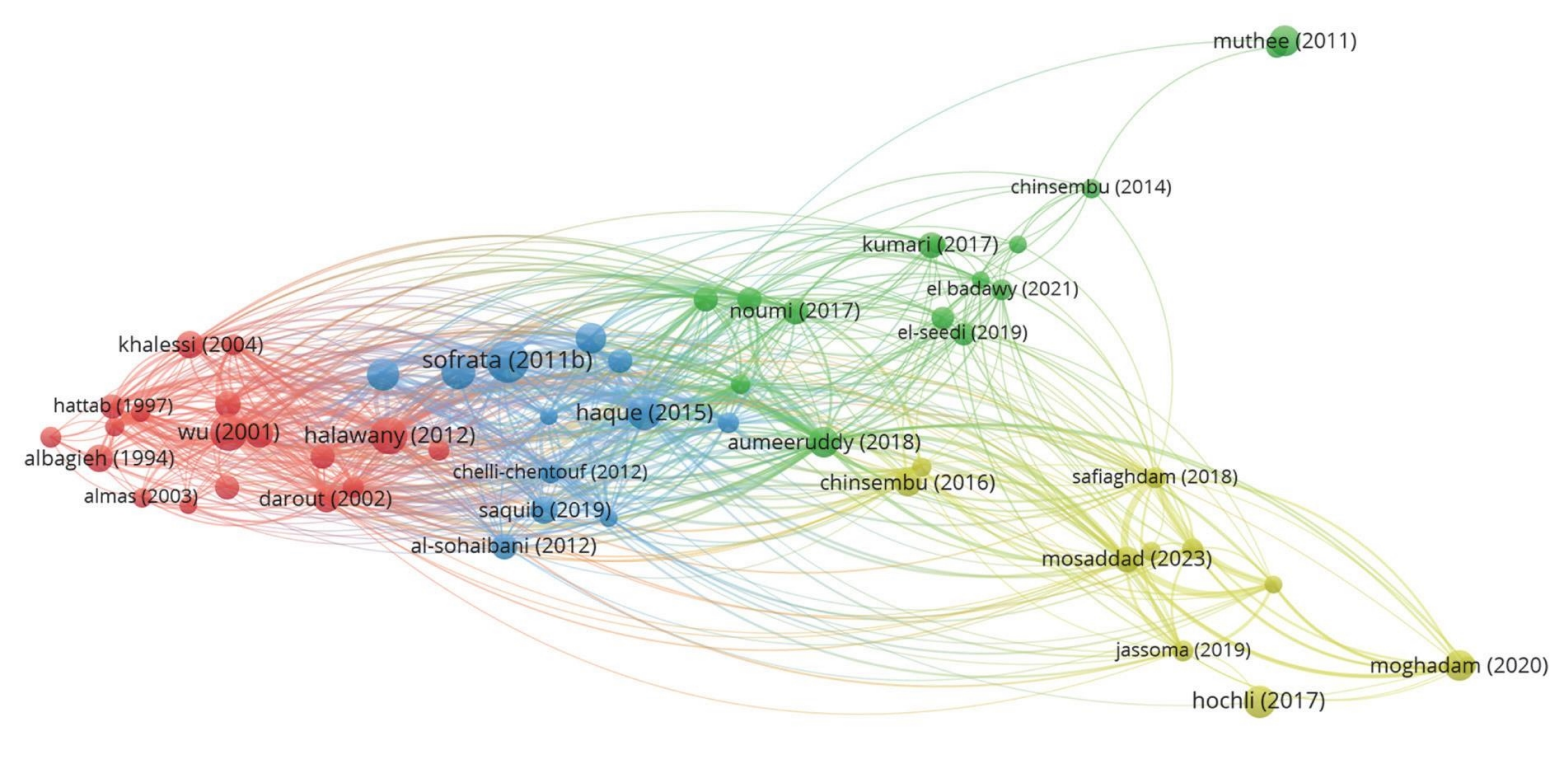
Bibliographic coupling network of documents in *Salvadora persica* oral health research.

To generate the bibliographic coupling map, VOSviewer was used with a minimum citation threshold of 59, yielding 27 eligible documents. This threshold was selected after repeated testing to balance clarity and coverage; extreme thresholds may exclude important studies or include redundant links, resulting in dense maps [15, 24]. The resulting network creates four distinct, overlapping clusters (Figure 6), suggesting common reference points and an overall structure of interrelated research topics. The map shows that documents on *S. persica* oral health research are organised into thematically related groups based on shared references. These groups reflect different research orientations, including oral hygiene use, antimicrobial activity, phytochemical characterisation and natural product oral health applications. Bibliographic coupling indicates the interrelationships among citing documents; therefore, total link strength (TLS) was the main measure of connectivity. TLS represents the cumulative strength of the links between one item and all other items in the network. In bibliographic coupling, a link is formed when two documents share references. Therefore, a document with a higher TLS has stronger overall bibliographic connections with other documents in the network, indicating that it shares more references, or more strongly connected reference patterns, with the rest of the mapped literature. The 10 documents with the highest TLS values are summarised in Table 3.

**Table 3:** Top documents identified by bibliographic coupling based on total link strength.

Document	Citation	Total link strength
Halawany [7]	114	356
Haqueand Alsareii [3]	95	350
Wu [18]	118	276
Aumeeruddy [6]	82	243
Sofrata [17]	39	158
Mosaddad et al. [25]	60	157
Sofrata [21]	57	125

#### 3.5.1. Cluster 1 (Red): Public Health, Oral Hygiene Behaviour and Clinical Use of Miswak

Research within this theme collectively highlights the role of miswak (*S. persica*) in oral hygiene from the public health, oral hygiene behaviour and clinical perspectives. El Bcheraoui et al. [26] reported limited oral health practices and a low prevalence of preventive dental services among the Saudi Arabian population, highlighting the urgency of implementing oral health promotion programmes in primary healthcare centres in this area [26]. It is against this background that Halawany et al. [7] examined the past application of miswak in the area, the chemical composition, and the possible advantages and drawbacks of miswak in the context of oral health, which places it in the context of why it is of interest to the present preventive dentistry [7]. In line with these reports, Wu et al. [18] also showed equivalent efficacy of chewing a stick and toothpaste in plaque removal due to mechanical effects, increased salivary flow, and an antimicrobial effect when used properly. According to the authors, the World Health Organisation also acknowledges that chewing sticks have the potential to enhance oral health, but additional scientific testing is required. The combination of these studies informs the role of miswak as a culturally relevant, bioactive and possibly effective oral health agent. The value of incorporating traditional practices into evidence‐based oral health promotion strategies is also emphasised [18].

#### 3.5.2. Cluster 2 (Blue): Antimicrobial Mechanisms and Therapeutic Potential of *S. persica*

Studies in this cluster report on the antimicrobial activity and the mechanistic basis of *S. persica* in controlling oral disease‐causing pathogens. According to Sofrata et al. [17], benzyl isothiocyanate is the major compound in *S. persica* roots and is among the volatile compounds that exhibit bactericidal activity against Gram‐negative pathogens. The mechanistic evidence was supported by chemical characterisation and ultrastructural analysis [17]. In the meantime, Al Bayati [27] reported that the aqueous and methanol extracts of *S. persica* exhibit broad‐spectrum antimicrobial activity against major oral microorganisms and *Candida albicans*. The streptococci were particularly susceptible [27]. In larger research, Haque and Alsareii [3] showed that miswak had antibacterial, antifungal, antiviral, antiplaque and anticariogenic effects,and provided clinical studies comparing the mechanical and chemical actions of miswak with conventional toothbrushing. Recently, Saquib et al. [28] demonstrated the therapeutic potential of *S. persica* due to its synergistic antibacterial activity, with plant extracts and antibiotics interacting to be effective against periodontal pathobionts, making miswak an adjunct to existing antimicrobial therapy. Collectively, these studies present *S. persica* as a well‐characterised, promising biologically active agent with adjunct applications in oral hygiene and periodontal therapy.

#### 3.5.3. Cluster 3 (Green): Phytochemical Characterisation, Biofunctional Properties and Translational Potential of *S. persica*

In this context, the application of *S. persica* extends beyond oral hygiene devices to encompass its phytochemical, biofunctional, and translational capabilities. Aumeeruddy et al. [6] have synthesised both traditional and modern evidence to display the pharmacological relevance of *S. persica* [6]. Developing this biochemical approach, Kumari et al. [29] have embarked on nutraceutical profiling of *S. persica* fruit, revealing sugars, essential minerals, amino acids, antioxidants and bioactive metabolites and evaluating their applicability as functional foods and sources of pharmaceutical compounds [29]. In the meantime, Noumi et al. [30] reported the anti‐quorum‐sensing and antibiofilm effects of miswak methanolic extracts on oral Staphylococcus species, suggesting a new non‐antibiotic approach to treating biofilm‐related oral diseases [30]. Lastly, El‐Seedi et al. [31] documented the historical medical relevance of *S. persica* in Traditional Islamic Medicine, underscoring the need to mediate traditional uses with clinically tested applications [31]. Collectively, these studies frame *S. persica* as an important bioresource that is rich in phytochemicals, bioactivity (antimicrobial and antibiofilm), nutraceutical value and translational potential.

#### 3.5.4. Cluster 4 (Yellow): Natural‐Product Oral Healthcare and Contextual Positioning of *S. persica*

*S. persica* belongs to a bigger category of natural and herbal products that are utilised in the promotion of oral health and in the treatment of diseases. Mosaddad et al. [25] underscore the worldwide prevalence of periodontal diseases and dental caries,and their connection to the oral microbiota, biofilm generation and oral inflammation. The issue of antimicrobial resistance and the general side effects of traditional agents are also mentioned, as is the possibility of plant‐based alternatives to overcome the issue [25]. In a similar vein, Moghadam et al. [23] conducted a thorough review of herbal medicines with oral health therapeutic potential, and, in terms of accessibility, lower cost, reduced side effects, and efficacy, the authors feel that herbal medicines are gaining acceptance [23]. Clinically, Laleman et al. [32] reported growing scientific and popular interest in natural products to support periodontal health, given their effectiveness in preventive and therapeutic dentistry, although the researchers also indicate the weaknesses of evidence because of limited sample sizes, limited study durations, and unstandardised formulations in the existing literature [32]. Chinsembu et al. [33] studied the geographical and ethnobotanical range of *S. persica*, reporting regional research trends, including sourcing in Africa and product development in Asia and South America [33]. Collectively, these studies indicate the potential of natural and herbal products, including miswak, to oral care and the necessity of further studies, including clinical trials, safety assessments and guidelines, to help incorporate them into dental practice.

In general, the bibliographic coupling analysis reveals that research on *S. persica* in oral health and dental hygiene developed in a sequence, starting with basic research, followed by mechanistic validation, translational research and clinical application.

### 3.6. Keyword Co‐Occurrence Analysis

**Figure 7:**
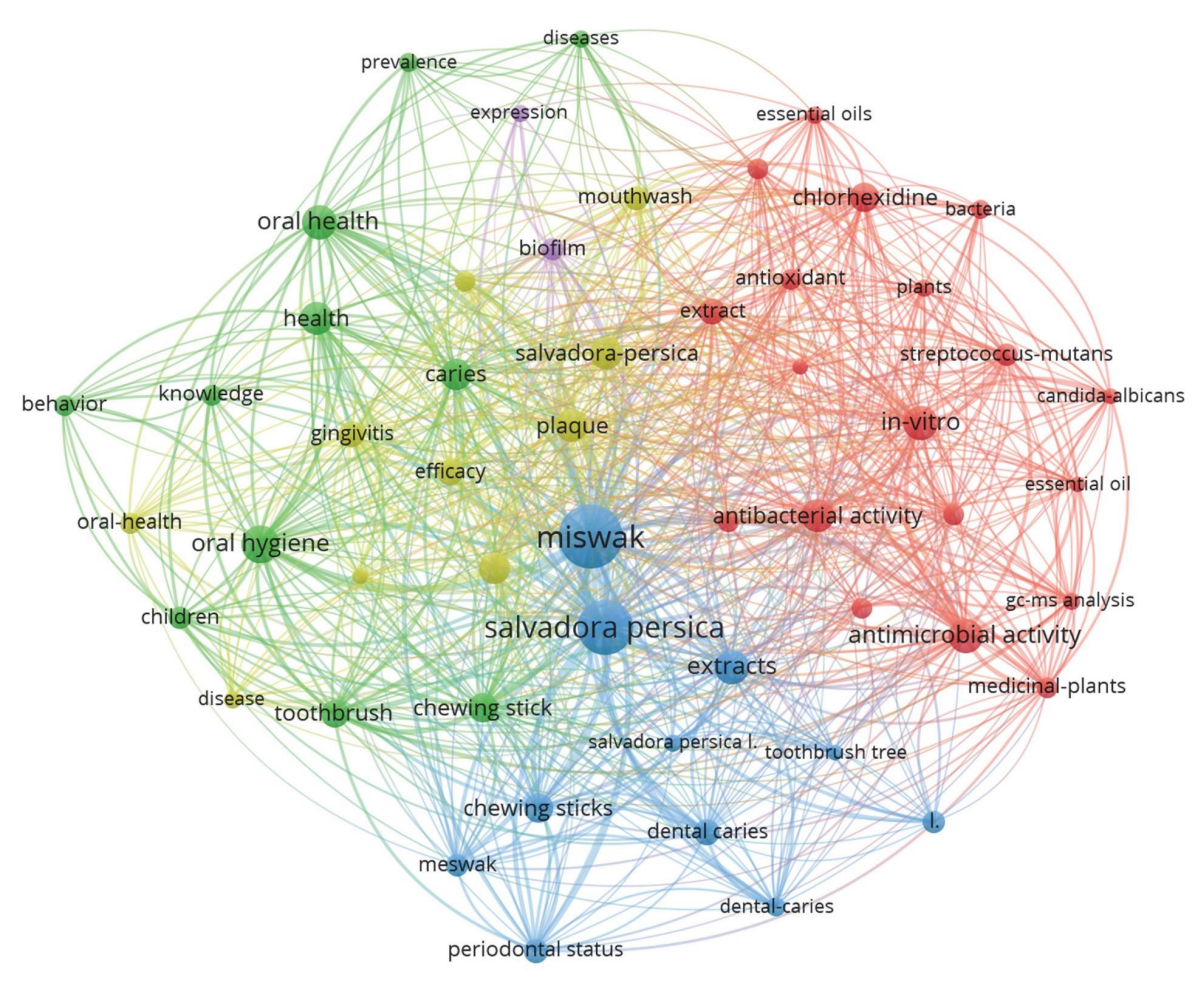
Keyword co‐occurrence network of *Salvadora persica* in oral health research.

A minimum keyword occurrence threshold of 5 was used, yielding 53 keywords in the final network. There were four thematic clusters that can be considered the conceptual framework for research on *S. persica* and oral health (Figure 7).

“Miswak” and “*Salvadora persica*” emerged as the most important terms, ranking the highest for occurrence and TLS (Table 4). The strong interrelationship between the two terms is evident in the current literature on scientific characterisation and traditional identity. Other keywords closely related include plaque, oral hygiene, and chewing stick, indicating their interest in preventive oral hygiene measures and plaque management. Extracts, antimicrobial activity, antibacterial activity, and in vitro testing were also quite common, demonstrating the application of laboratory‐based assessment to the mechanistic authentication of clinical observations on *S. persica*. Lastly, the names *Streptococcus mutans*, *Candida albicans*, and dental caries indicate research on oral pathogens with cariogenic and periodontal significance. Overall, high keyword frequency and strong TLS point to the integration of traditional oral hygiene practices into experimental antimicrobial research.

**Table 4:** Most frequent keywords and network connectivity in *Salvadora persica* oral health research based on co‐word analysis.

Keyword	Occurrences	Total link strength
Miswak	102	394
*Salvadora persica*	77	236
Plaque	26	137
Extracts	29	130
Antimicrobial activity	29	129
Antibacterial activity	27	120
In vitro	30	120
Oral hygiene	35	119
Chewing stick	22	109
Salvadora‐ persica	26	105

#### 3.6.1. Cluster 1 (Red): Antimicrobial and Antioxidant Mechanisms of *S. persica*

This cluster uses keywords such as antibacterial activity, antioxidant, bacteria, and *C. albicans* to determine the *biological and mechanistic properties* of *S. persica*. In vitro investigations assessing the antimicrobial efficacy against oral pathogens predominate in this cluster. Isolated extracts with antioxidant and pathogen‐inhibitory effects, contributing to research interest in the potential biological activity of *S. persica*.

#### 3.6.2. Cluster 2 (Green): Behavioural, Preventive and Population‐Based Oral Health Contexts

Keywords such as *behaviour*, *children*, *caries*, *disease* and *chewing stick* make up this cluster. There is a focus on oral hygiene practices, behavioural determinants and preventive outcomes with the use of miswak in different populations. These results are compared with trends and prevalence of oral disease, with an emphasis on epidemiology and the community. The cluster discusses the significance of behaviour and education in introducing miswak use in oral healthcare practice, particularly in the context of public health and preventive dentistry.

#### 3.6.3. Cluster 3 (Blue): Clinical and Extract‐Based Applications of Miswak in Dental Caries Management

Keywords in this cluster are miswak, chewing sticks, extracts and caries. The articles within this group are related to formulation and clinical relevance. Alternative systems of delivery, such as crude extracts and modified systems, are evaluated based on their effectiveness in caries and their associated outcomes. The traditional and clinically derived terms used suggest the translation of the traditional oral hygiene idea into the modern clinical context in preventive dental care research.

#### 3.6.4. Cluster 4 (Yellow): Plaque Control and Gingival Health Outcomes

The keywords defining this cluster are dental plaque, gingivitis, efficacy and mouthwash, implying that the studies directly establish clinical outcomes in oral disease. In this topic, the research typically compares products made from miswak or *S. persica* with traditional oral hygiene products, such as toothbrushes and chlorhexidine mouthwashes. This cluster reflects research interest in plaque control and gingival health outcomes associated with *S. persica*‐based interventions. However, because this is a bibliometric analysis, these patterns should not be interpreted as direct evidence of clinical efficacy.

## 4. Implications

The present bibliometric analysis provides an overview of how studies on *S. persica* (miswak) are organised and developed across the literature on oral hygiene. Thematic clusters were employed to identify specific progress in this area of research: the shift from culturally based, descriptive research to more complex, mechanistic, translational, and clinically based research. The microbiology, phytochemistry, and preventive dentistry themes form the foundation for assessing miswak as a mechanical plaque remover and an antimicrobial agent. This paper combines traditional and modern sciences to emphasise the importance of miswak as a topic in preventive oral health research and to underscore that its role is not limited to its ethnomedical and cultural history.

At present, formal evidence‐based clinical guidelines for the use of miswak or *S. persica*‐based products in dental practice remain limited. This absence represents an important implementation gap. Therefore, clinicians should interpret the available literature cautiously and avoid treating bibliometric patterns as proof of clinical effectiveness. Evidence‐based guidance can be used by dental hygienists and oral health educators during preventive counselling to inform indications and techniques. Finally, geographically centred research could be used at the public health level to align oral health promotion strategies with local cultures. Nevertheless, professional standards, product quality control and clinical evidence, rather than informal use, must be upheld before adoption into routine practice.

## 5. Limitations and Future Research

Several limitations were identified during this study. First, this bibliometric analysis was limited to the records indexed in the WoS Core Collection. Although WoS provides curated bibliographic metadata, citation information and cited references suitable for bibliometric mapping, the use of a single database may have excluded relevant studies indexed only in Scopus, PubMed, Google Scholar or regional databases. This is particularly important for oral health, ethnomedicine and plant‐based dental research, where some biomedical, regional, non‐English or recently published articles may be indexed outside WoS. Therefore, the findings should be interpreted as a WoS‐indexed representation of *S. persica* oral health research rather than a complete coverage of all available literature. Future bibliometric studies may combine WoS with Scopus, PubMed or other databases to provide broader coverage and enable database‐comparison analysis. Second, while the approach provides information on research trends, influence, and thematic structure, it offers no insight into study quality or clinical effectiveness. Furthermore, the citation impact can be misinterpreted as proof of method superiority. Additionally, variations in terminology may have affected retrieval. Relevant studies may refer to *Salvadora persica* using alternative terms such as miswak, siwak, meswak, chewing stick, toothbrush tree, plant extract or herbal oral‐care agent. Some studies may also discuss *S. persica* without including these terms in the title, abstract or keywords, which could limit retrieval through topic‐based searching. Finally, the publication year affects citation‐based indicators, potentially underrepresenting more recent clinical studies.

Methodologically robust clinical trials should be conducted in future research, focusing on measuring clinically relevant outcomes such as plaque control, oral health, and patient‐reported measures using standardised miswak preparations. Other measurable outcomes, such as long‐term safety, side effects and comparisons with established oral hygiene products, should be included. Furthermore, complex mechanistic studies of the oral microbiome and biofilm‐focused approaches could elucidate novel biological pathways. Finally, research on population behaviour, including compliance, user practices, access and cultural acceptability, should be conducted before widespread implementation.

## 6. Conclusion

This bibliometric analysis provides a structured overview of *research on S. persica oral healt*h indexed in the WoS Core Collection. The findings show that the literature is organised around oral hygiene practices, antimicrobial and phytochemical mechanisms, clinical oral health outcomes, and broader applications of natural products. However, bibliometric patterns should not be interpreted as direct evidence of clinical effectiveness. The study highlights the need for standardised terminology, reproducible experimental protocols, stronger clinical study designs, long‐term safety evaluation and clearer implementation frameworks before *S. persica*‐based products can be incorporated into evidence‐based dental recommendations.

## Author Contributions

**Norfaezah Ahmad:** conceptualisation, investigation, writing – original draft; writing – review and editing. **Muhammad Ashraf Fauzi:** conceptualisation, methodology, software, formal analysis, data curation, visualisation, supervision, writing – review and editing. **Nur Armidha Nadihah:** investigation, data curation, writing – original draft, writing – review and editing. **Nurulhasanah Mustapar:** investigation, resources, writing – review and editing. **Sharifah Nurul Akilah Syed Mohamad:** investigation, data curation, writing – review and editing. **Mohd Hafiz Arzmi:** conceptualisation, supervision, project administration, validation, writing – review and editing.

## Funding

This work was funded by the Ministry of Higher Education, Malaysia (Grant FRGS/1/2023/SKK11/UIAM/03/1) and International Islamic University Malaysia (Grant CHAIN 24‐010‐0010). Open access publishing facilitated by the University of Melbourne, as part of the Wiley — The University of Melbourne agreement via the Council of Australian University Librarians.

## Disclosure

All authors have read and approved the final version of the manuscript. Mohd Hafiz Arzmi, as the corresponding author and Muhammad Ashraf Fauzi, as the co‐corresponding author and the manuscript guarantor, had full access to all of the data in this study and take complete responsibility for the integrity of the data and the accuracy of the data analysis. All references were checked for retractions and corrections. No cited reference was found to be retracted at the time of resubmission.

## Ethics Statement

This study did not require ethical approval because it was a bibliometric analysis based entirely on bibliographic records retrieved from the Web of Science Core Collection. No human participants, biological samples, patient records, personal identifiers, clinical images or animal subjects were involved.

## Conflicts of Interest

The authors declare no conflicts of interest.

## Data Availability

The authors confirm that the data supporting the findings of this study are available within the article. The bibliographic dataset analysed in this study was retrieved from the Web of Science Core Collection using the search strategy and screening procedure described in the Methods section.
